# Reproducibility of macular ganglion cell–inner plexiform layer thickness in normal eyes determined by two different OCT scanning protocols

**DOI:** 10.1186/s12886-017-0434-2

**Published:** 2017-04-04

**Authors:** Xiaoyu Xu, Xinxing Guo, Hui Xiao, Lan Mi, Xiangxi Chen, Xing Liu

**Affiliations:** State Key Laboratory of Ophthalmology, Zhongshan Ophthalmic Center, Sun Yat-sen University, 54 South Xianlie Road, Yuexiu District, Guangzhou, Guangdong 510060 People’s Republic of China

**Keywords:** Optical coherence tomography, Ganglion cell-inner plexiform layer, Reproducibility, Protocol

## Abstract

**Background:**

To investigate the reproducibility of macular ganglion cell-inner plexiform layer (GCIPL) thickness measurement in normal eyes determined by different operators and two different raster scanning protocols of Cirrus high-definition optical coherence tomography (HD-OCT).

**Methods:**

One hundred and two eyes of 102 normal subjects were scanned three times using Cirrus HD-OCT with Macular Cube 512 × 128 protocol by two operators, respectively. Three extra scans were obtained using Macular Cube 200 × 200 protocol. The average, minimum, superotemporal, superior, superonasal, inferonasal, inferior, and inferotemporal GCIPL thickness was measured. The reproducibility of the measurements was evaluated with intraclass correlation coefficients (ICC) and coefficients of variation (CoV).

**Results:**

The intra-operator ICCs of macular GCIPL thickness were >0.875; and the inter-operator ICCs were 0.882 to 0.991. The intra-protocol ICCs of Macular Cube 512 × 128 and 200 × 200 protocol were 0.953 to 0.987 and 0.953 to 0.991, respectively; and the inter-protocol ICCs were 0.876 to 0.991. All CoVs were <1.5%.

**Conclusions:**

Cirrus HD-OCT can measure macular GCIPL thickness in normal eyes with excellent reproducibility. The measurements determined by Macular Cube 512 × 128 and 200 × 200 protocol were highly consistent and both protocols were eligible for macular GCIPL thickness measurement.

## Background

Glaucoma ranks first in diseases causing irreversible blindness worldwide [1]. The introduction of spectral-domain optical coherence tomography (SD-OCT) has enabled in-vivo structural and quantitative assessment of the optic nerve head (ONH) and peripapillary retinal nerve fiber layer (RNFL) thickness with good precision and reproducibility, which is a crucial component in glaucoma diagnosis and management [2].

A number of studies have shown that structural changes of glaucoma primarily affect retinal ganglion cells (RGC) and their axons [3]. SD-OCT is able to image the macular area, where the highest concentration of ganglion cells locates. The enhanced scan resolution, better quality image acquisition, and more accurate automatic segmentation of retinal layers make the measurement of the macular ganglion cell-inner plexiform layer (GCIPL) thickness possible [4]. One of the commercially available OCT devices that can measure GCIPL thickness is Cirrus high-definition optical coherence tomography (HD-OCT; Carl Zeiss Meditec, Dublin, CA).

Several studies have demonstrated that Cirrus HD-OCT can measure GCIPL thickness with excellent reproducibility in both normal and glaucomatous subjects [5–7]. Notably, Cirrus HD-OCT has two macular volume scanning protocols, Macula Cube 200 × 200 and Macula Cube 512 × 128, and the images taken by both protocols can be analyzed by the ganglion cell analysis (GCA) algorithm. The difference between the two raster scanning protocols is the number of B-scans that cover a retinal volume. The official recommended scanning protocol for the GCIPL thickness analysis is Macula Cube 200 × 200. However, Macula Cube 512 × 128 scanning protocol has a denser B-scan than Macula Cube 200 × 200, thus may generate a more precise result. Hagen et al. confirmed that both protocols showed excellent reproducibility of retinal thickness and volume measures [8]. However, significant differences between protocols for retinal thickness in several subfields were found. In that case, they recommended to use Macula Cube 512 × 128 protocol instead of the 200 × 200 protocol in retinal thickness and retinal volume measurements. To the best of our knowledge, the comparison of the reproducibility between the two macular scanning protocols have not yet been reported. The purpose of the present study therefore, was to evaluate the intra- and inter-operator, and the intra- and inter-protocol reproducibility of macular GCIPL thickness measurement using Cirrus HD-OCT in Chinese ethnicity.

## Methods

### Participants

This study complied with the Declaration of Helsinki and was approved by the Institutional Review Board of Zhongshan Ophthalmic Center of Sun Yat-sen University. Written informed consent was obtained from all participants. Participants enrolled in this study were recruited from volunteers who agreed to undergo the examinations and met the eligibility criteria described below at Zhongshan Ophthalmic Center, Sun Yat-sen University, Guangzhou, China between April 2013 to March 2015.

All participants underwent complete ophthalmic evaluation including examinations of uncorrected and best-corrected visual acuity, refraction examination, intraocular pressure (IOP) measurement using a Goldman applanation tonometer, slit lamp biomicroscopy examination, gonioscopy, dilated fundus examination, stereo disc photography (Kowa nonmyd a-D III; Kowa Optimed Inc., Aichi, Japan); standard automated perimetry using the SITA standard 30–2 program (Humphrey Visual Field Analyzer; Carl Zeiss Meditec, Inc.); and SD-OCT examination (Cirrus HD-OCT; Carl Zeiss Meditec, Dublin, CA).

For inclusion, the criteria were: (i) age ≥ 18 years; (ii) best-corrected visual acuity 20/20 or better; (iii) a spherical equivalent refractive error between −6 diopters (D) and +2 D; (iv) intraocular pressure ≤ 21 mmHg; (v) normal optic nerve head (cup-to-disc ratio < 0.5 in either eye, cup-to disc ratio asymmetry ≤0.2, no evidence of optic disc hemorrhage or focal thinning of the rim) and macula appearance by dilated stereoscopic examination and fundus photography; (vi) reliable and normal visual field testing; (vii) normal open anterior chamber angle; (viii) normal retinal nerve fiber layer (RFNL) thickness on OCT analyzer. Exclusion criteria were: (i) history of glaucoma, uveitis, vitreoretinal diseases or nonglaucomatous optic neuropathy; (ii) evidence of media opacities; (iii) prior intraocular surgery or ocular trauma; (iv) neurological or systemic diseases that could affect retina and visual field results, including dementia or multiple sclerosis; (v) debilitating or life-threatening diseases. When data from both eyes were eligible for analysis, only one randomly selected eye from each patient was included in this study.

### Optical coherence tomography imaging

A junior operator (XX, Operator A) and a senior operator (HX, Operator B) performed OCT scanning on qualifying eyes dilated with tropicamide 1% and phenylephrine 2.5% (Mydrin®-P, Santen Pharmaceutical Co. Ltd., Osaka, Japan) with the same Cirrus HD-OCT device, respectively. The Cirrus HD-OCT offers two different macular volume scanning protocols within a cube measuring 6 × 6 × 2 mm centered at the foveal: Macular Cube 200 × 200 scanning protocol (200 horizontal B-scans comprising 200 A-scan per B-scan), and Macular Cube 512 × 128 scanning protocol (128 horizontal B-scans comprising 512 A-scan per B-scan). At least 3 scans were obtained using Macular Cube 512 × 128 scanning protocol by each operator at the same visit. At least 3 additional scans were obtained using Macular Cube 200 × 200 scanning protocol by Operator A. A 5-min interval between each scan was guaranteed and artificial tear was provided if dryness or dazzle was complained by the participants. Images with signal strength <6 and those with visible eye motion or blinking artifacts (discontinuous jump) were considered of poor quality and discarded immediately during the image acquisition process.

The ganglion cell analysis (GCA) algorithm (Cirrus Version 6.0; Carl Zeiss Meditec, Dublin, CA) was able to identify the outer boundary of the RNFL and the inner plexiform layer (IPL) so that the distance between the RNFL and the IPL outer boundary segmentations yields the combined thickness of the ganglion cell layer (GCL) and IPL (termed “GCIPL”). The following GCIPL thickness were analyzed: average, minimum, and sectoral (superior, superonasal, inferonasal, inferior, inferotemporal, and superotemporal). The minimum GCIPL thickness is defined as the minimum measurement of the 1-degree interval by sampling 360 spokes extending from the center of the fovea to the edge of the ellipse. For peripapillary RNFL thickness, the average, superior, temporal, inferior and nasal quadrant thicknesses were analyzed by the Cirrus internal analysis algorithm.

### Statistical analysis

Statistical analyses were performed using SPSS version 20.0 (SPSS Inc., Chicago, IL). Kolmogorov-Smirov test and Levene test were conducted to test the normality and homogeneity of variance, respectively. The repeated measurements conducted by each operator and each protocol of GCIPL thickness were compared using analysis of variance (ANOVA). The differences of the above-mentioned parameters between the two operators and the two protocols were compared using the Student’s *t*-test. Reproducibility was assessed with the coefficient of variation (CoV) and the intraclass correlation coefficient (ICC) with 95% confidence interval (CI). The intra-operator and intra-protocol ICC was calculated with the three repeated measurements, and the inter-operator and inter-protocol ICC was calculated with a randomly-selected measurement from each operator or each protocol. The correlation between mean signal strength and CoV of three repeated measurements obtained by each scanning protocol was investigated using Pearson’s correlation coefficient. A *P* value <0.05 was considered statistically significant.

## Results

A total of 108 participants were enrolled in the study based on the inclusion criteria. Of these, 6 participants were excluded because of segmentation failure of the OCT images. Thus, the analyses in this study were based on data from 102 subjects (52 males and 50 females). The subjects ranged in age from 19 to 75 years (mean, 46.0 ± 16.2 years). The IOP ranged from 9 to 19 mmHg (14.3 ± 2.5 mmHg). The spherical equivalent of refractive error ranged from −5.25 to +2.00 D (−0.57 ± 1.88 D). The mean deviation of the visual field testing ranged from −2.05 to 1.74 dB (−0.77 ± 0.84 dB).

Mean values of the average, minimum, superior, superonasal, inferonasal, inferior, inferotemporal, and superotemporal macular GCIPL thickness measured by the two operators were displayed in Table 1. There was no significant difference of the three repeated measurements of the GCIPL thickness parameters by each operator, and the difference of the GCIPL thickness measured by the two operators also showed no statistical significance (all *P* value >0.05). Table 2 showed the intra- and inter- operator ICC for GCIPL thickness measurements, of which the highest and lowest records were the average GCIPL thickness and minimum GCIPL thickness, respectively.

**Table 1:** GCIPL thickness (Mean ± SD, μm) measured by two operators using macular cube 512 × 128 scanning protocol

Parameter	Operator	1st Measurement	2nd Measurement	3rd Measurement	*P* ^a^	*P* ^b^
Average	A	84.33 ± 5.76	84.27 ± 5.54	84.23 ± 5.63	0.991	0.846
	B	84.38 ± 5.58	84.42 ± 5.59	84.39 ± 5.64	0.999	
Minimum	A	81.44 ± 5.61	81.65 ± 5.30	81.23 ± 5.76	0.859	0.850
	B	81.79 ± 5.72	81.71 ± 5.69	81.16 ± 5.97	0.689	
Superotemporal	A	82.88 ± 5.29	82.82 ± 5.20	82.80 ± 5.42	0.993	0.938
	B	82.74 ± 5.41	82.73 ± 5.36	82.71 ± 5.35	0.999	
Superior	A	85.48 ± 6.15	85.33 ± 5.90	85.38 ± 5.99	0.983	0.935
	B	85.31 ± 5.88	85.31 ± 5.89	85.41 ± 5.93	0.992	
Superonasal	A	87.06 ± 6.57	86.86 ± 6.40	86.77 ± 6.56	0.943	0.966
	B	86.76 ± 6.37	86.88 ± 6.30	86.82 ± 6.43	0.990	
Inferonasal	A	84.94 ± 6.46	84.96 ± 6.24	84.91 ± 6.06	0.998	0.847
	B	85.29 ± 6.13	85.17 ± 6.11	85.34 ± 6.39	0.978	
Inferior	A	82.42 ± 6.25	82.44 ± 6.03	82.30 ± 6.07	0.982	0.792
	B	82.72 ± 6.20	82.73 ± 6.29	82.79 ± 6.38	0.996	
Inferotemporal	A	83.27 ± 5.81	83.38 ± 5.67	83.34 ± 5.86	0.990	0.916
	B	83.46 ± 5.68	83.56 ± 5.82	83.53 ± 5.91	0.991	

^a^comparison between the three repeated measurements of the GCIPL thickness measured by each operator

^b^comparison of the GCIPL thickness measured by the two operators

**Table 2:** Intra- and inter- operator reproducibility of GCIPL thickness measurements

	Operator A			Operator B			Inter-operator		
Parameter	ICC	95% CI	CoV (%)	ICC	95% CI	CoV (%)	ICC	95% CI	CoV (%)
Average	0.986	0.981–0.990	0.55	0.992	0.989–0.994	0.44	0.991	0.987–0.994	0.47
Minimum	0.887	0.849–0.918	1.28	0.875	0.833–0.909	1.37	0.882	0.827–0.919	1.24
Superotemporal	0.963	0.949–0.973	0.94	0.977	0.969–0.984	0.79	0.967	0.952–0.977	0.73
Superior	0.956	0.940–0.968	1.08	0.983	0.977–0.988	0.78	0.978	0.968–0.985	0.88
Superonasal	0.953	0.936–0.966	0.95	0.983	0.976–0.988	0.65	0.948	0.925–0.964	0.71
Inferonasal	0.956	0.940–0.968	0.93	0.973	0.964–0.981	0.86	0.973	0.961–0.982	0.81
Inferior	0.960	0.946–0.971	1.15	0.959	0.944–0.971	1.16	0.961	0.944–0.973	1.01
Inferotemporal	0.981	0.973–0.986	0.81	0.979	0.971–0.985	0.80	0.977	0.967–0.984	0.81

*ICC* intraclass correlation coefficient, *CI* confidence interval, *CoV* coefficients of variation

Mean values of the average, minimum and sectorial GCIPL thickness determined by Macular Cube 512 × 128 and Macular Cube 200 × 200 scanning protocols were displayed in Table 3. There was no significant difference of the three repeated measurements of the GCIPL thickness determined by each protocol, and the differences of the GCIPL thickness determined by the two protocols also showed no statistical significance (all *P* value >0.05). However, the average signal strength of Macular Cube 512 × 128 and Macular Cube 200 × 200 scanning protocols was 8.54 ± 1.13 and 7.60 ± 1.06, respectively. The difference between each protocol was statistically significant (*t* = 10.618,*P* < 0.001). The intra-protocol ICC of the two protocols and the inter-protocol ICC ranged between 0.953–0.987, 0.953–0.991 and 0.876–0.991, respectively (Table 4). The correlations of mean signal strength and CoV of three repeated measurements obtained by each scanning protocol were displayed in Table 5. A significant positive correlation was found in average (*r* = −0.261 and −0.210, respectively) and minimum GCIPL thickness (*r* = −0.243 and −0.372, respectively) obtained by both protocols, inferonasal GCIPL thickness obtained by Macular Cube 512 × 128 protocol (*r* = −0.280), and superotemporal (*r* = −0.208), inferior (*r* = −0.293), and inferotemporal GCIPL thickness (*r* = −0.288) obtained by Macular Cube 200 × 200 protocol.

**Table 3:** GCIPL thickness (Mean ± SD, μm) determined by two macular cube scanning protocols

Parameter	Protocol	1st measurement	2nd measurement	3rd measurement	*P* ^a^	*P* ^b^
Average	512 × 128	84.65 ± 5.79	84.50 ± 5.63	84.46 ± 5.65	0.984	0.892
	200 × 200	84.61 ± 5.71	84.70 ± 5.69	84.72 ± 5.61	0.994	
Minimum	512 × 128	81.80 ± 5.33	81.72 ± 5.13	81.43 ± 5.25	0.927	0.632
	200 × 200	81.72 ± 5.87	81.70 ± 5.73	80.91 ± 6.28	0.722	
Superotemporal	512 × 128	83.04 ± 5.06	82.76 ± 5.11	82.85 ± 5.21	0.96	0.909
	200 × 200	82.67 ± 5.40	82.87 ± 5.37	82.70 ± 5.05	0.977	
Superior	512 × 128	85.83 ± 6.10	85.67 ± 6.22	86.00 ± 6.07	0.961	0.974
	200 × 200	85.72 ± 6.03	85.80 ± 6.03	85.79 ± 5.98	0.997	
Superonasal	512 × 128	87.96 ± 6.56	87.72 ± 6.24	87.83 ± 6.15	0.981	0.963
	200 × 200	87.81 ± 6.39	87.83 ± 6.35	87.92 ± 6.27	0.995	
Inferonasal	512 × 128	85.22 ± 6.33	85.11 ± 6.24	84.94 ± 6.12	0.973	0.772
	200 × 200	85.52 ± 6.22	85.35 ± 6.17	85.70 ± 6.30	0.96	
Inferior	512 × 128	82.50 ± 6.32	82.48 ± 6.07	82.20 ± 6.36	0.963	0.726
	200 × 200	82.54 ± 6.51	82.61 ± 6.28	83.00 ± 6.52	0.923	
Inferotemporal	512 × 128	83.46 ± 5.77	83.39 ± 5.58	83.28 ± 5.72	0.986	0.973
	200 × 200	83.35 ± 5.62	83.52 ± 5.69	83.42 ± 5.64	0.988	

^a^Comparison between the three repeated measurements of the GCIPL thickness determined by each raster scanning protocol

^b^Comparison of the GCIPL thickness determined by the two raster scanning protocols

**Table 4:** Intra- and inter- protocol reproducibility of GCIPL thickness measurements

	Macular Cube 512 × 128 Protocol			Macular Cube 200 × 200 Protocol			Inter-protocol		
Parameter	ICC	95% CI	CoV (%)	ICC	95% CI	CoV (%)	ICC	95% CI	CoV (%)
Average	0.987	0.980–0.992	0.58	0.991	0.986–0.995	0.39	0.991	0.984–0.995	0.44
Minimum	0.960	0.938–0.975	1.47	0.951	0.924–0.970	1.04	0.876	0.786–0.928	1.45
Superotemporal	0.955	0.930–0.972	0.89	0.971	0.955–0.982	0.67	0.942	0.902–0.966	0.94
Superior	0.957	0.934–0.974	1.14	0.985	0.976–0.991	0.81	0.983	0.970–0.990	0.61
Superonasal	0.970	0.953–0.981	1.06	0.990	0.984–0.994	0.74	0.987	0.978–0.993	0.48
Inferonasal	0.979	0.968–0.987	1.02	0.980	0.968–0.988	0.88	0.983	0.971–0.990	0.75
Inferior	0.953	0.928–0.971	1.12	0.953	0.928–0.971	1.07	0.960	0.932–0.977	1.10
Inferotemporal	0.978	0.965–0.986	0.77	0.974	0.960–0.984	0.73	0.973	0.953–0.984	0.92

*ICC* intraclass correlation coefficient, *CI* confidence interval, *CoV* coefficients of variation

**Table 5:** The correlation of mean signal strength and coefficient of variation (CoV) of repeated measurements of GCIPL thickness obtained by two macular cube scanning protocols

	Macula Cube 512 × 128 Protocol		Macula Cube 200 × 200 Protocol	
Parameter	*r* ^a^	*P*	*r* ^a^	*P*
Average	−0.261	0.008	−0.210	0.034
Minimum	−0.243	0.014	−0.372	<0.001
Superotemporal	−0.179	0.072	−0.208	0.036
Superior	−0.064	0.525	0.029	0.774
Superonasal	−0.135	0.176	−0.043	0.669
Inferonasal	−0.280	0.004	−0.186	0.062
Inferior	0.025	0.805	−0.293	0.003
Inferotemporal	−0.049	0.627	−0.288	0.003

^a^Pearson’s correlation coefficient

## Discussion

In this study, we evaluated the intra- and inter-operator reproducibility of macular GCIPL thickness measurement and investigated the reproducibility of the two available raster scanning protocols of the Cirrus HD-OCT in a Chinese population. Generally, we confirmed that the Cirrus HD-OCT could measure macular GCIPL thickness in normal eyes with excellent reproducibility.

In our study, all ICCs of the macular GCIPL thicknesses ranged between 0.875 and 0.992, and the CoVs were <1.5%. The ICCs were highest for the average GCIPL and lowest for the minimum GCIPL thickness. These findings were in line with the previous findings. Choi et al. accessed the reproducibility of macular GCIPL thicknesses in 44 glaucoma patients. The ICCs were between 0.96 and 0.99, and the CoVs were <3% [5]. In 50 glaucoma patients, Mwanza et al. found that all intervisit ICCs ranged between 0.94 and 0.98 and the CoVs were <5% [6].

Our results showed that nearly all intra- and inter-operator ICCs were greater than 0.90, suggesting that the measurements were hardly influenced by the operators’ experiences. Given the greater image resolution, higher acquisition speed, and identical scanning location with better macular segmentation algorithms, it is not unexpected to see highly reproducible results of the repeated measurements. With the high speed camera and the improvements of Cirrus software like FoveaFinder™, AutoCenter™, and FastTrac™ retinal tracking system, eye motion artifacts can be reduced and images captured in identical locations from visit to visit can be guaranteed [9]. Moreover, high reproducibility may also due to real-time removal of unqualified images. The reproducibility of minimum GCIPL thickness measurement was slightly lower than other parameters. It is possibly because that other GCIPL parameters were yielded from data equalization of 60 degree (sectoral) or 360 degree (average), while the minimum GCIPL thickness was calculated from data of 1-degree interval with the lowest average of the 360 spokes of the ellipse, where bias was more likely to happen.

Notably, studies on GCIPL thickness measurements using Cirrus HD-OCT were mostly based on the Macular Cube 200 × 200 scanning protocol [7, 10, 11]. The reproducibility and possible differences of GCIPL thickness determined by Macular Cube 512 × 128 and 200 × 200 scanning protocols were still unknown. We found that there was no significant difference of GCIPL thickness measured by the two protocols. All intra- and inter-protocol ICCs were greater than 0.876, and all ICCs other than the minimum GCIPL thickness were greater than 0.94, which demonstrated that the two protocols were highly consistent and both protocols were eligible for macular GCIPL thickness measurement.

The protocols differed in definition, but both protocols were able to calculate the macular GCIPL thickness within the same 14.13mm^2^ elliptical annulus area (dimensions: a vertical inner and outer radius of 0.5 mm and 2.0 mm and a horizontal inner and outer radius of 0.6 and 2.4 mm, respectively) centered on the fovea. The size of the inner ring in the annulus was chosen to exclude the foveal area where the ganglion cell layer is too thin to detect; the size and shape of the outer ring was selected because it corresponds closely to the area where the RGCs are densest in real anatomy. Macular Cube 512 × 128 scanning protocol was supposed to have higher reproducibility than Macular Cube 200 × 200 protocol due to higher definition and better image acquisition. However, the reproducibility of the former was not superior to that of the latter in our study. Reasons may be that higher definition could result in longer duration time (2.4 s VS 1.3 s), making the former more influenced by factors including poorer quality of tear film, eye motion or blinking, thus offset part of the benefits brought by higher resolution of the images.

OCT measures GCIPL thickness by defining the inner boundary of ganglion cell layer and the outer boundary of inner plexiform layer by means of detecting the differences of reflectivity between various retinal layers. In a scan with higher signal strength, light penetrates deeper into the retina and increases the reflectivity of all layers, thus more tissues would have a reflectivity higher than the detection threshold of OCT devices [12, 13]. Wu et al. demonstrated that scans with signal strength of at least 7 had higher reproducibility and were associated with greater RNFL thickness in Stratus OCT. [14] Zhang et al. found that the repeatability of RNFL and GCC thickness measurements may be improved by excluding images with weak signal strength below recommended cutoffs of signal strength index of a Fourier-domain OCT device [15]. In our study, the signal strength of Macular Cube 512 × 128 and Macular Cube 200 × 200 protocol were (8.54 ± 1.13) and (7.60 ± 1.06), respectively. The statistical significance was found in signal strength between the two protocols while the GCIPL thickness measured by the two protocols were of no statistical significance. Therefore, signal strength of at least 7, which is a proxy for scan quality guarantee, should be achieved, regardless of which protocol is used for GCIPL thickness measurement. The correlation between mean signal strength and CoV of the repeated measurements obtained by Macular Cube 200 × 200 protocol was more significant than Macular Cube 512 × 128 protocol. An increased variability of the measurements is expected to be associated with lower signal strength. The most possible reason may be that the lower signal strength of the former disclosed the tendency. In a scale of relatively higher signal strength, the signal strength and the thickness measurement would be less relevant, which is consistent with previous results [14, 16, 17]. Another reason may be that the higher scanning density of Macular Cube 512 × 128 protocol compensate the variation caused by the decrease in signal strength in a certain extent.

There were several limitations of our study. First, we enrolled only healthy volunteers in OCT imaging with pupil dilated, and we discarded unqualified OCT scans and scans with signal strength less than 6 immediately according to the real-time monitoring when taking OCT examinations. However, scans with borderline qualification are almost inevitable in a clinical setting rather than an experimental setting. Moreover, previous studies demonstrated that the reproducibility of GCC thickness measurements in glaucomatous patients was inferior to that of normal subjects. In this case, a comprehensive evaluation of the reproducibility of GCIPL thickness measurement should include both normal and abnormal population with a relatively easier eligibility criteria of the OCT scans which mimic the real clinical setting. Second, the difference of the signal strength of the two protocols may be due to the defective imaging procedure. In our study, at least 3 scans were obtained using Macular Cube 512 × 128 scanning protocol, followed by another 3 scans using Macular Cube 200 × 200 protocol. Although break and artificial tear were provided, it was difficult to eliminate the impacts of patient fatigue, possible decline of stability of tear film, and increased likelihood of fixation loss during the examination.

## Conclusions

In conclusion, our study demonstrated that both macular cube scanning protocols of the Cirrus OCT provided satisfactory reproducibility for GCIPL thickness measurement in Chinese population. The results of the measurement were hardly influenced by the operators’ experiences. Moreover, we also found that Macular Cube 200 × 200 scanning protocol would be less time-consuming than Macular Cube 512 × 128 protocol without sacrificing the reproducibility and scanning quality and reproducibility when the signal strength was greater than 7. We therefore recommend to use the 200 × 200 protocol in measuring the macular GCIPL thickness when the signal strength of at least 7 can be achieved.

## Abbreviations

CI: Confidence interval
CoV: Coefficients of variation
GCA: Ganglion cell analysis
GCIPL: Ganglion cell-inner plexiform layer
GCL: Ganglion cell layer
HD-OCT: High-definition optical coherence tomography
ICC: Intraclass correlation coefficient
IOP: Intraocular pressure
IPL: Inner plexiform layer
ONH: Optic nerve head
RGC: Retinal ganglion cells
RNFL: Retinal nerve fiber layer
SD-OCT: Spectral-domain optical coherence tomography
